# Risk Factors for *Brucella* Seroprevalence in Peri-Urban Dairy Farms in Five Indian Cities

**DOI:** 10.3390/tropicalmed4020070

**Published:** 2019-04-22

**Authors:** Johanna F. Lindahl, Jatinder Paul Singh Gill, Razibuddin Ahmed Hazarika, Nadeem Mohamed Fairoze, Jasbir S. Bedi, Ian Dohoo, Abhimanyu Singh Chauhan, Delia Grace, Manish Kakkar

**Affiliations:** 1 Department of Biosciences, International Livestock Research Institute, Nairobi 00100, Kenya; J.lindahl@cgiar.org (J.F.L.); D.Randolph@cgiar.org (D.G.); 2 Department of Clinical Sciences, Swedish University of Agricultural Sciences, PO Box 7054, SE-750 07 Uppsala, Sweden; 3 Zoonosis Science Centre, Department of Medical Biochemistry and Microbiology, Uppsala University, Po Box 582, SE-751 23 Uppsala, Sweden; 4 Guru Angad Dev Veterinary and Animal Sciences University, Ludhiana 141004, Punjab, India; gilljps@gmail.com (J.P.S.G.); bedijasbir78@gmail.com (J.S.B.); 5 Department of Veterinary Public Health, Assam Agricultural University, Khanapara Campus, Guwahati-781022, India; rah1962@rediffmail.com; 6 Department of LPT, Veterinary College, Karnataka Veterinary Animal & Fisheries Sciences University Bangalore, Bangalore 560024, India; prof.nadeem@gmail.com; 7 Atlantic Veterinary College, University of Prince Edward Island, Charlottetown, C1A 4P3, Canada; dohoo@upei.ca; 8 Public Health Foundation India, Gurgaon 122002, India; abhimanyu.hm@gmail.com; 9 Department of Public Health Sciences, Faculty of Medicine, University of Liège, 4000 Liege, Belgium

**Keywords:** zoonoses, prevalence, *Brucella abortus*, urban livestock keeping, smallholder farming

## Abstract

Brucellosis is endemic among dairy animals in India, contributing to production losses and posing a health risk to people, especially farmers and others in close contact with dairy animals or their products. Growing urban populations demand increased milk supplies, resulting in intensifying dairy production at the peri-urban fringe. Peri-urban dairying is under-studied but has implications for disease transmission, both positive and negative. In this cross-sectional study, five Indian cities were selected to represent different geographies and urbanization extent. Around each, we randomly selected 34 peri-urban villages, and in each village three smallholder dairy farms (defined as having a maximum of 10 dairy animals) were randomly selected. The farmers were interviewed, and milk samples were taken from up to three animals. These were tested using a commercial ELISA for antibodies against *Brucella abortus*, and factors associated with herd seroprevalence were identified. In all, 164 out of 1163 cows (14.1%, 95% CI 12.2–16.2%) were seropositive for *Brucella*. In total, 91 out of 510 farms (17.8%, 95% CI 14.6–21.4%) had at least one positive animal, and out of these, just seven farmers stated that they had vaccinated against brucellosis. In four cities, the farm-level seroprevalence ranged between 1.4–5.2%, while the fifth city had a seroprevalence of 72.5%. This city had larger, zero-grazing herds, used artificial insemination to a much higher degree, replaced their animals by purchasing from their neighbors, were less likely to contact a veterinarian in case of sick animals, and were also judged to be less clean. Within the high-prevalence city, farms were at higher risk of being infected if they had a young owner and if they were judged less clean. In the low-prevalence cities, no risk factors could be identified. In conclusion, this study has identified that a city can have a high burden of infected animals in the peri-urban areas, but that seroprevalence is strongly influenced by the husbandry system. Increased intensification can be associated with increased risk, and thus the practices associated with this, such as artificial insemination, are also associated with increased risk. These results may be important to identify high-risk areas for prioritizing interventions and for policy decisions influencing the structure and development of the dairy industry.

## 1. Introduction

Infectious diseases cause a major burden on both human health and society as a whole. Zoonotic diseases inflict a double burden, since they also affect animal health, with associated costs and reduced productivity. Brucellosis is a very common but frequently neglected zoonosis that occurs globally, except for in a few countries that have managed to eradicate it, and the disease is often underreported and uncontrolled in low and middle-income countries, which may have the highest burden of the disease [1,2,3,4].

The disease can be caused by different bacteria of the genus *Brucella*, of which most species are pathogenic to multiple mammals, including cattle and humans [5,6]. Cattle are most frequently infected by *Brucella abortus* or *Brucella melitensis* [5,6]. These bacteria cause a chronic infection with preferred localization in the reproductive system, where they can cause abortion in pregnant cows or other reproductive problems, as well as reduced milk production in lactating animals and orchitis in bulls [1,7]. Although most infected animals only abort once, they may remain infected their entire life [8]. After the first abortion, as well as in non-pregnant animals, the disease can be asymptomatic [5]. Infected male cattle can spread the disease sexually, and both sexes may become infertile. Joint hygromas are another common manifestation of brucellosis [3]. 

Milk consumption has been increasing in low and middle-income countries, a trend likely to continue as the demand for animal-source food trends upwards due to population growth, changing lifestyles, and increasing wealth [9]. In India, the large vegetarian population increases the dependence on dairy products for high quality proteins. India has the world’s largest dairy herd at around 300 million and is the world’s leading milk producer, contributing around 17% of the world’s total milk production, with more than 70 million households engaged in milk production [10,11]. Milk consumption is higher in urban areas and while the majority of the Indian population still live in rural areas, urbanization is increasing. Cities require a constant supply of fresh milk, and peri-urban dairy production plays an important role in meeting this demand. 

The health of livestock, humans and livelihoods are closely linked, with zoonotic diseases such as brucellosis causing not only human and animal morbidity, but also reduced animal production and hence reduced incomes [12,13]. In India, awareness of brucellosis is low among livestock-keepers and healthcare staff, and because of the non-specific symptoms and the limited availability of laboratory facilities in many rural hospitals, diagnosis is seldom feasible [14,15]. Multiple studies have found seropositivity in humans in India, indicating the need to have an OneHealth approach for controlling this disease [16].

## 2. Materials and Methods

### 2.1. Ethical Approval

The study received ethical approval from the ethics committee of the Public Health Foundation of India [TRC-IEC-219/14, 27 May 2014; amended 12 October 2015]. Ethical approval was also obtained by Institutional Ethics Committees of Guru Angad Dev Veterinary and Animal Sciences University (GADVASU), Assam Agricultural University (AAU), Karnataka Veterinary, Animal and Fisheries Sciences University (KVAFSU), Rajasthan University of Veterinary and Animal Sciences (RAJUVAS) and School of Biotechnology, Kalinga Institute of Industrial Technology (KSBT) at the Ludhiana, Guwahati, Bangalore, Udaipur and Bhubaneswar study sites, respectively. Before a farmer was interviewed, they were informed about the purpose of the study and gave their consent to participate.

### 2.2. Farm Selection

**Figure 1:**
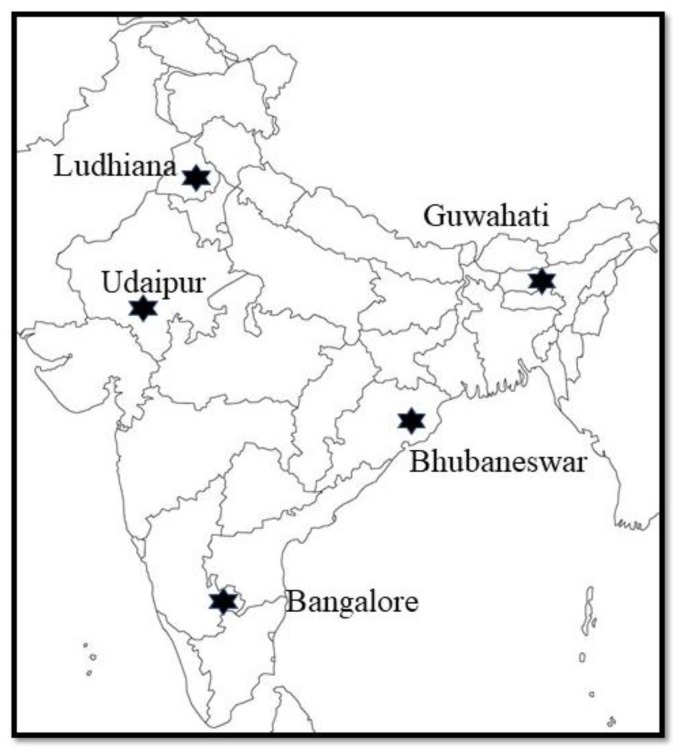
Location of selected cities in India.

Five Indian cities were selected purposively to represent different parts of the country (Figure 1). Peri-urban was defined as within 5 km of the official city boundaries, and all villages in that circle were mapped. For the purpose of this study, smallholder farms were defined as a dairy farm with a herd size of less than 10 cattle/buffaloes at the time of the survey and at least one milking animal, with dairy constituting a source of livelihood with or without domestic consumption. A systematic selection of 34 of these villages was done by identifying the proportion that needed to be sampled, and then systematically choosing these in a clockwise fashion around the city. The selected villages were then visited to identify all farms, using local village leaders as guides. The methodology of the creation of this sampling frame has been described elsewhere [17]. Out of this sampling frame, three smallholder farmers per village were randomly selected. 

### 2.3. Data Collection

Data collection was done between June 2015 and January 2016. A questionnaire was developed and piloted on farms in each site before starting the sampling. The tool was uploaded into electronic format and data collection was conducted using tablets, from which the data was uploaded into a central server. Data was collected by different data collection teams in each city, but all teams were trained by the same trainers, who joined in the first days of data collection. The data was collected through interviews in the local language, after the participants had been read the information about the project and given their written consent. Observations about cleaning practices and hygienic status were done during milking using an observation checklist. Cleanliness and drainage scores were standardized using pictures to guide the grading, and the scoring was assessed during the training to make sure it was consistent. Knowledge about antibiotics was assessed based on if the farmer reported to know the word (in the local language).

### 2.4. Sample Collection

In each farm, up to three milking cows or buffaloes were selected for sampling. In the 33% of farms where the number of milking cattle exceeded three, all cows were given a unique number, and then three numbers were selected randomly. The data collection teams were trained on aseptic collection of milk from the selected cows, and 40 mL of milk was collected in sterile vials and immediately kept chilled until they were transported to the laboratory on the same day of collection. The samples were thereafter stored in deep freezer at −80 °C. Samples were kept frozen when transported to the laboratory of microbiology at GADVASU and stored at −80 °C until analysis. 

### 2.5. Serological Analysis

Milk was analyzed for the presence of antibodies using a commercial indirect enzyme-linked immunosorbent assay (iELISA) developed for use with milk samples (IDEXX Brucellosis Milk X2 Ab Test, IDEXX, Westbrook, ME, USA). The protocol of the manufacturer was followed, and all samples were done in duplicates. In brief, 50 µL of milk was diluted into 200 µL of sample diluent on a microplate precoated with *Brucella* lipopolysaccharide (LPS). The plate was incubated for 90 min, before being washed and the subsequent addition of conjugated anti-bovine IgG antibodies. After 30 min of incubation and washing, the tetramethylbenzidine (TMB) substrate was added and incubated for 20 min before the stop solution was added and the plate read at 450 nm. 

The ratio of the optical density of the samples (mean) to the mean positive control was calculated after subtracting the mean of the negative controls from both. A ratio of above 55% was considered positive, while between 45 and 55% was considered suspected positive. In the analysis, only one sample was suspected positive, and since all other animals tested at the same farm were also positive, this animal is considered positive in the analyses and results. The specificity for this kit used on milk samples has been found to be very high [18], and a meta-analysis suggest a specificity of 96% for ELISA conducted on milk [19]. 

### 2.6. Data Analysis

**Figure 2:**
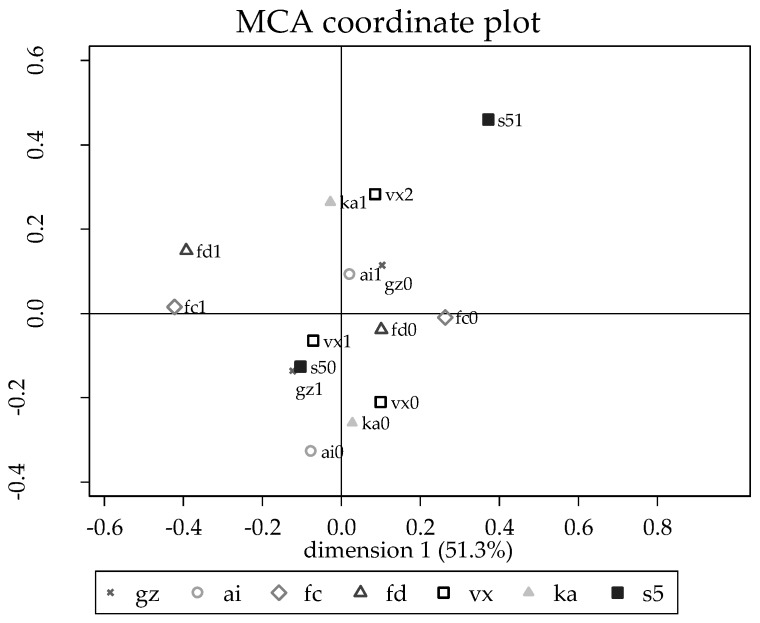
Multiple correspondence analysis (MCA) plot for the factors associated with the high seroprevalence site. Gz = grazing, where gz0 is zero-grazing and gz1 is grazing. Ai = artificial insemination, where ai0 is no artificial insemination and ai1 is use of artificial insemination. Fc = floor cleanliness, where fc0 is clean or moderately clean floor and fc1 is dirty floor. Fd = drainage, where fd0 equals insufficient drainage and fd1 is good drainage. Vx = vaccination, where vx0 means no vaccination done, vx1 means vaccination when there is an outbreak or when given free vaccines, and vx2 means vaccinating animals as young. Ka = knowledge about antibiotics, where ka0 is no knowledge and ka1 is the farmer reporting to know about antibiotics. S5 = site Guwahati, where s50 means any other site and s51 means Guwahati.

An initial screening of all information collected was carried out with only the variables listed in Table 1 being retained for analysis, after identifying the variables with potential causal association with brucellosis. Analysis of the retained data proceeded in four steps. First, multiple correspondence analysis (MCA) was used to investigate relationships among all the predictors recorded. All predictors were initially recoded to di- or trichotomous variables. An MCA was carried out using all predictors and those having a contribution to either the first or second dimension that exceeded 0.02 were retained. The process was repeated sequentially with the required contribution being raised by 0.01 at each step (to a maximum of 0.06). At this point, the six variables that best explained the information content of the full set had been identified and these were plotted on a two-dimensional MCA plot (Figure 2).

One site (Guwahati) had a dramatically higher farm prevalence (72%) than the other four sites (4%), so the second step in the analysis was to evaluate the unconditional associations between each of the predictors and a variable representing Guwahati compared to the other sites. Either two-sample *t*-tests or cross-tabulations with chi-square statistics were used to determine if the regions were different. Results are presented in Table 1. Guwahati was also added as a supplemental variable (s51 vs s50 in Figure 2) to the MCA plot in Figure 2 to show which predictors were most associated with Guwahati vs other sites.

The third step was to use logistic regression models to identify factors associated with the risk of a farm being *Brucella* spp. positive within Guwahati, using backward elimination among variables with unconditional associations with *p* < 0.15. A random effect for village was included in all models. The linearity of continuous predictors was evaluated using lowess smoothed curves and a quadratic term was added to the model if there was significant evidence of curvature in the relationship. Initially, unconditional associations were determined with predictors having *p* < 0.15 retained for further consideration. A manual backward elimination was used to remove non-significant (at *p* > 0.05) predictors. Age of farmer and farm size (number of animals) were forced into all models as potential confounders. In addition to age and number of animals, two factors (cleanliness of floor and level of vaccination) were identified as potentially important. An MCA plot was generated (using trichotomous versions of each of the predictors) with *Brucella* spp. added as a supplemental variable to the final plot to see which predictors were generally associated with being *Brucella* spp. positive or negative.

Finally, the model building process was repeated to determine which factors most influenced the risk of being *Brucella* spp. positive in sites 1 to 4. Only two predictors (farm size and use of *Haemorrhagic septicemia* vaccine) had unconditional associations with *p* < 0.15, but neither of these was significant in a final model so no results are presented. 

## 3. Results

### 3.1. Brucella Seroprevalence

In total, 164 out of 1163 cows (14.1%, 95% CI 12.2–16.2%) were seropositive for *Brucella*. 

A farm was considered positive for *Brucella* if at least one out of the three tested animals tested positive. In total, 91 farms out of 510 (17.8%, 95% CI 14.6%–21.4%) had at least one positive animal (see Table 2), and out of these, 23 farms had two positive animals and 25 (all in Guwahati) had all three animals positive. There were large differences in farm prevalence between the five different cities (Table 2). Guwahati had significantly higher seroprevalence (*p* < 0.001) than the other sites, and the odds ratio for a farm being positive in Guwahati was 44.4 (95% CI 7.5–113.2) times higher than in Udaipur and 138.9 (95% CI 32.0–602.3) times higher than in Bhubaneswar.

### 3.2. Risk Factor Analyses for Herds with No Previous Vaccination

The presence of different stipulated risk factors varied across the five cities. After exclusion of farms that reported having vaccinated against *Brucella* earlier, 460 farms from 162 villages in five sites (geographic regions) were included. Missing values were observed in 0 to 15.6% of observations within a variable and 62% of farms had complete data for all variables. *Brucella* prevalence ranged from 1.4 to 5.2% across four sites, while the prevalence in Guwahati was 72.5%.

The MCA analysis for investigating relationships among predictors identified pasture grazing, use of artificial insemination (AI) (vs natural breeding), routine (vs irregular) vaccination, floor cleanliness, adequacy of floor drainage, and owner knowledge of antibiotics as the six variables most useful in discriminating among farms. Floor cleanliness and drainage contributed most to the first dimension (explaining 51.3% of inertia (information) in the data) while level of vaccination, knowledge of antibiotics and AI contributed most to the second dimension (45.0% of inertia). Farmers that had knowledge of antibiotics also used routine vaccination, and they tended to be farms that did not pasture (graze) animals but did use AI. Farms with good floor cleanliness also had good floor drainage.

Evaluation of unconditional associations between the recorded predictors and Guwahati vs the other sites showed many statistically significant differences. With the exception of three of the four demographic variables and the quarantining of new entries into the herd, all predictors showed significant differences at *p* < 0.05. Compared to the other cities, Guwahati had larger non-pastured herds, used AI for breeding, purchased their replacements from neighbors, were less likely to have good stable cleanliness or drainage scores, were more likely to have dirty stable floors, had a lower composite hygiene score, and were less likely to have veterinarians regularly check their animals or check animals before purchase. However, they were more likely to use routine vaccinations of young animals and to know what antibiotics were.

### 3.3. Risk Factors for Brucella Seropositivity in Guwahati

**Figure 3:**
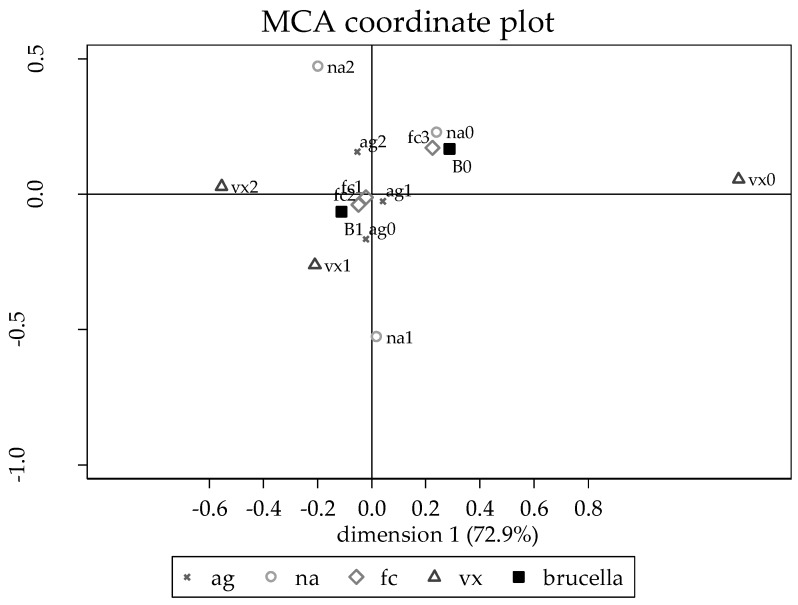
MCA plot of risk factors for *Brucella* seropositivity in Guwahati, India. Ai = Artificial insemination, where ai0 is no artificial insemination and ai1 is use of artificial insemination. Na = number of animals, where na0 is less than 7 animals and na1 is 7–10 animals. Fc = floor cleanliness, where fc0 is clean or moderately clean floor and fc1 is dirty floor. Vx = vaccination, where vx0 means no vaccination done, vx1 means vaccination when there is an outbreak or when given free vaccines, and vx2 means vaccinating animals as young. B = *Brucella*, where B0 = seronegative farm and B1 = seropositive farm.

In addition to age and herd size, which were included as potential confounders, two management factors (floor cleanliness and level of vaccine use) were identified as being associated with *Brucella* spp. A multiple correspondence analysis (MCA) plot was generated (using trichotomous versions of each of the predictors) with *Brucella* spp. added as a supplemental variable to the final plot to see which predictors were generally associated with being *Brucella* spp. positive or negative (Figure 3). Being *Brucella* positive (B1) was most common in farms that had a younger age owner (ag0 or ag1) and had a lower floor cleanliness scores (fc1 or fc2). Being *Brucella* negative (B0) was most strongly associated with the cleanest floors (fc3) and the smallest herds (na0). Table 3 shows the odds ratios associated with seropositivity for risk factors in Guwahati from the multivariable model. The high village level variance indicates a very high intra-cluster correlation. 

### 3.4. Risk Factors for Brucella Seropositivity in Bangalore, Bhubaneswar, Ludhiana, and Udaipur

Logistic models were also used to investigate risk factors for *Brucella* spp. positivity within the low-prevalence sites. However, no risk factors had significant associations, so no results are presented.

## 4. Discussion

This study found high variation in the seroprevalence between the different peri-urban sites. In general, our findings were comparable with the literature on bovine brucellosis in India. A recent review concluded that most studies using probabilistic sampling and not targeting cows with a clinical history suggesting brucellosis, reported a prevalence of 5–12%, which is above what was detected in the four other cities but considerably less than that we found in Guwahati [20]. While this study could not identify many risk factors in the peri-urban farms, it was found that keeping floors clean was important. Risk of *Brucella* exposure was also associated with herd size, which has been shown previously [21,22,23,24]. Vaccinating (for other diseases than brucellosis), either routinely or when there were vaccination campaigns in the face of outbreaks or vaccines provided for free, was associated with a higher risk of exposure, which could potentially be explained as farmers with more experience with disease being more positive to vaccination. 

The peri-urban seroprevalence in Ludhiana, Punjab, was lower than previously reported in the state. Here 21% and 18% seroprevalence was found by Aulakh et al. [25] and Ul-Islam et al. [26]. Gill et al. [23] and Dhand et al. [27] also found more than 10% prevalence in cattle. This may indicate that reducing prevalence is associated with better control or changing husbandry, or may reflect a more systematic approach to sampling in our study, which focused only on smallholder peri-urban dairying.

The high seropositivity in Guwahati is in accordance with results from the same area using a milk ring test, where 88% of farms were found positive [28]. Chakraborty et al. [29] found 60% seropositivity among lactating cows in Guwahati, whereas Gogoi et al. [30] found 30% seroprevalence in Kamrup metropolitan district of Guwahati. It is worth noting that Renukaradhya et al. [31] did not find any seropositive animals in Assam, using their own developed ELISA. Given the results of our research and the earlier research from the state, it seems that the peri-urban belt around Guwahati may have a higher than average burden of brucellosis. Brucellosis in humans has not been extensively studied, but in one previous study, three (all with animal husbandry background) out of 52 humans tested positive in Assam [32]. In people with animal contact, more than 24% seropositivity was found in Ludhiana as well [33], indicating the need to include brucellosis as a potential diagnosis in febrile cases with occupational risk factors. 

The study shows how MCA can be used when data are collected on quite a large number of predictors and many of these are potentially related (weakly or strongly). In this context, it is useful to visualize these inter-relationships in order to get a better understanding of how farms could be grouped. The MCA identified a set of key variables which could be used to discriminate among farms, and these should be considered as important to collect information on in any future research undertaken in this region of India. Another methodological issue was presented because one site (Guwahati) had an extremely high herd prevalence of *Brucella* spp. compared to the other four sites. The strong collinearity between the outcome of interest (seropositivity against *Brucella*) and the site meant that the risk factor analyses could not utilize the full data set in one analysis. It would have been impossible to tell if any significant predictor was actually associated with *Brucella* spp. positivity or whether it just strongly differed between Guwahati and the other sites (with no effect on *Brucella* spp. risk). Consequently, risk factors were evaluated in Guwahati separately from the other study sites, which reduced the power of the study. For the four low-prevalence sites, there were only 14 positive farms (out of 362) while in Guwahati there were only 27 negative farms (out of 98). This lack of power limited our ability to identify risk factors for *Brucella* spp.

Raising awareness, training farmers, and modern techniques are often recommended for improving livestock disease control. In our study, the evidence for this was ambiguous. Guwahati, which had the highest prevalence in this study, was characterized by greater knowledge and higher use of modern animal health care inputs, such as vaccination and AI. On the other hand, hygiene appeared to be poor. Overall, a picture emerges of larger, less well managed herds with more reliance on vaccines and antibiotics for disease control. Other studies in Guwahati found that while training interventions had some impact on both hygiene and knowledge, there was no impact on the seropositivity for brucellosis [28]. This indicates caution in assuming intensification, even with improving knowledge and training, will lead to better disease control. It should be noted, however, that overall use of vaccination was low, indicating considerable scope for improvement. There seemed to be a high dependency on vaccinations in the face of outbreaks or when they were provided for free, with two of the sites having less than 3% of farms reporting routine vaccinations. Poor vaccination coverage can have different explanations, including poor access to vaccines, limited extension services, or poor understanding of farmers as to the benefits of using vaccines. In other studies in India, low knowledge about the function of vaccines and low willingness to pay has been associated with low uptake of vaccination [34,35], which could possibly also explain the low adoption here. Studying the use of vaccines in chickens, it has been shown that having active support promotes vaccination and also makes people understand the function of vaccines better, which makes for more positive attitudes, and hence better uptake [36]. Even though farms that reported having used *Brucella* vaccines were excluded from analyses, it is possible that there might be farms where the farmer did not know which disease the animals were vaccinated against, which could have affected the results, but considering the low vaccination against *Brucella* overall, this is deemed a low risk. India has a government sponsored control program for brucellosis in cattle, with planned use of the S19 vaccine [31], but still vaccination is seldom performed in the field. 

Many sero-surveys have been carried out for brucellosis in India, but these are typically conducted in one area, and differing methods make it hard to compare results from different areas, including the frequent targeting of animals with clinical symptoms [20]. Using the same, probabilistic study approach contemporaneously in five widely dispersed cities allowed us to confidently detect important and likely real differences between cities and to link this with some risk factors. An important finding of the study was that brucellosis can be very prevalent in some peri-urban areas and have very low presence in others. Moreover, disease transmission risk factors are different in scenarios with a high or a low infection pressure, and a habit, such as purchasing cows from neighbors is likely a protective factor when living in a low-risk area, but a high-risk practice in an area with a very high prevalence. Within Guwahati, the mixed effects model suggested a very high village level variance and a high intra-cluster correlation, indicating that future studies need to include as many villages as possible, which could be explained by the habit of purchasing animals from nearby farms, spreading the disease within a village, but less so between villages.

## 5. Conclusions

This study emphasizes the need to systematically identify disease hotspots for zoonotic diseases; the importance of considering intensifying peri-urban dairy belts in disease surveillance and control; the high degree to which structural factors may influence disease risk in peri-urban dairy, and the need for targeted, effective interventions. In light of the brucellosis control program in India, this study highlights the lack of sufficient vaccination coverage among smallholder dairy farmers in different parts of India, and also the high variability in prevalence. Knowledge about the prevalence in different areas can guide the control efforts, and improved information about local risk factors as well as the extent of farmers’ understanding about the disease, can aid in creating better extension campaigns. 

## Figures and Tables

**Table 1:** Potential risk factors for brucellosis given as either mean (standard deviation), or proportion (95% confidence interval).

	All Sites	Guwahati	Bangalore	Bhubaneswar	Ludhiana	Udaipur	p-Value *
Age of farmer	46.2 (12.3)	43.9 (12.9)	44.8 (13.2)	51.1 (10.8)	46.6 (12.7)	44.6 (10.2)	0.035
Female respondents	21.8% (18.3–25.6)	15.7% (9.2–24.2)	41.2% (31.5–51.4)	11.8% (6.2–19.6)	14.7% (8.5–23.1)	25.5% (17.4–35.1)	0.096
Illiterate respondents	35.4% (31.0–39.9)	51.1% (40.2–61.9)	41.1% (30.8–52.0)	23.5% (15.7–33.0)	15.7% (8.6–25.3)	44.9% (34.8–55.3)	0.001
Number of dairy animals	7.7 (4.0)	10.3 (4.1)	5.4 (2.7)	8.1 (3.6)	7.2 (3.7)	7.5 (4.0)	<0.001
Zero-grazing	53.3% (48.9–57.7)	95.1% (88.9–98.3)	9.8% (4.8–17.2)	4.9% (1.6–11.1)	93.1% (86.4–97.2)	63.7% (53.6–73.0)	<0.001
Using AI	76.0% (72.1–79.7)	92.2% (85.1–96.6)	98.0% (93.1–99.8%)	47.5% (37.5–57.7)	69.6% (59.7–78.3)	72.5% (62.8–80.9)	<0.001
Purchasing cows from neighboring farms	57.6% (52.8–62.3)	98.9% (94.0–100)	71.7% (57.7–83.2)	21.1% (13.4–30.6)	34.4% (24.9–45.0)	69.0% (59.0–77.9)	<0.001
Dirty floors in cow sheds	11.1% (8.5–14.2)	24.8% (16.7–34.3)	11.0% (5.6–18.8)	10.8% (5.5–18.5)	6.1% (2.3–12.7)	3.0% (0.6–8.4)	<0.001
Well-drained floors	19.8% (16.4–23.6)	8.9% (4.2–16.2)	22.8% (15.2–32.5)	23.5% (15.7–33.0)	35.4% (26.0–45.6)	8.9% (4.2–16.2)	0.007
Never vaccinate animals	15.5% (12.4–18.9)	25.5% (17.4–35.1)	0% (0–3.6)	2.0% (0.2–6.9)	7.8% (3.4–14.9)	42.42% (32.4–52.3)	<0.001
Vaccinate young animals routinely	26.7% (22.9–30.7)	61.8% (51.6–71.2)	2.9% (0.6–8.4)	44.1% (34.3–54.3)	0% (0–3.6)	24.5% (16.5–34.0)	<0.001
Records of sick animals	11.6% (8.0–14.7)	0% (0–3.6)	8.8% (4.1–16.1)	24.5% (16.5–34.0)	1.0% (0–5.3)	23.5% (15.7–33.0)	<0.001
Alpha score for cleaning routines	2.19 (0.28)	2.14 (0.23)	2.10 (0.28)	2.16 (0.08)	2.06 (0.13)	2.50 (0.34)	0.034
Alpha score for observed hygiene	0.32 (0.28)	0.10 (0.11)	0.31 (0.21)	0.60 (0.31)	0.25 (0.21)	0.35 (0.23)	<0.001
Regular health checks	23.9% (20.3–27.9)	3.9% (1.1–9.7)	14.7% (8.5–23.1)	33.3% (24.3–43.4)	46.1% (36.2–56.2)	21.6% (14.0–30.8)	<0.001
Let veterinarian check animals before purchase, or test the animal	25.9% (22.1–29.9)	2.9% (0.6–8.4)	35.3% (26.1–45.4)	58.8% (48.6–68.5)	9.8% (4.8–17.3)	22.6% (14.9–31.9)	<0.001
Quarantine new animals	24.6% (20.8–28.4)	17.7% (10.8–26.4)	19.6% (12.4–28.6)	41.4% (31.6–51.8)	19.6% (12.4–28.6)	24.5% (16.5–34.0)	0.074

* Comparing Guwahati with the four other sites.

**Table 2:** Farm level *Brucella* seroprevalence (95% confidence interval) in the five different cities.

	*Brucella* Farm Positivity	*Brucella* Farm Positivity Excluding Farms with Vaccination
Bangalore	2.9% (0.6–8.4)	3.0% (0.6–8.6)
Bhubaneswar	2.0% (0.2–6.9)	1.4% (0–7.4)
Guwahati	73.5% (63.9–81.8)	72.5% (62.5–81.0)
Ludhiana	4.9% (1.6–11.1)	4.3% (1.2–10.6)
Udaipur	5.9% (2.2–12.4)	5.2% (1.7–11.6)
Overall	17.8% (14.6–21.34)	18.3% (14.8–22.1)

**Table 3:** Risk factors for *Brucella* seropositivity within Guwahati, India, using a mixed logistic regression model.

Risk Factor	Odds Ratio	95% Confidence Interval	Standard Error	*p*-Value
Farmer age (year)	0.96	0.90–1.02	0.03	0.20
Floor cleanliness				
Clean	Reference			
Average	11.6	1.29–105.18	13.1	0.03
Dirty	42.8	1.87–978.57	68.3	0.02
Vaccination				
No vaccination	Reference			
Vaccinate irregularly	44.1	0.73–2669.57	92.3	0.07
Vaccinate routinely	12.8	1.40–116.80	14.4	0.02
Number of animals	1.0	0.85–1.30	0.4	0.7
Quadratic term of number of animals	0.95	0.90–0.999	0.02	0.05
Constant	2.2	0.05–91.5	4.1	
Village level variance	4.2	0.65–26.84	4.0	
